# Relative Body Fat Distribution in Preadolescent Indian Children Exposed to a Natural Disaster during Early Development

**DOI:** 10.3390/ijerph19116356

**Published:** 2022-05-24

**Authors:** Aleksandra Gomula, Natalia Nowak-Szczepanska, Raja Chakraborty, Slawomir Koziel

**Affiliations:** 1 Department of Anthropology, Ludwik Hirszfeld Institute of Immunology and Experimental Therapy, Polish Academy of Sciences, 53-114 Wroclaw, Poland; natalia.nowak-szczepanska@hirszfeld.pl (N.N.-S.); slawomir.koziel@hirszfeld.pl (S.K.); 2 Department of Anthropology and Tribal Studies, Sidho-Kanho-Birsha University, Purulia 723104, West Bengal, India; rajanth2003@yahoo.co.uk; 3 Department of Anthropology, Dinabandhu Mahavidyalaya, Bongaon 743235, West Bengal, India

**Keywords:** adiposity distribution, central adiposity, natural disaster, prenatal development, infancy

## Abstract

Fetal life and infancy are critical periods when adverse environmental conditions, such as natural disasters, may alter a developing organism, leading to life-lasting unfavorable health outcomes, such as central body fat distribution. Therefore, the aim of this study was to assess the effect of the exposure to cyclone Aila in utero or during infancy on the relative subcutaneous adiposity distribution in preadolescent Indian children. The study included children prenatally (N = 336) or postnatally (during infancy, N = 212) exposed to Aila and a non-affected group (N = 284). Anthropometric indices involved, i.e., subscapular, suprailiac, triceps, and biceps skinfolds. The relative adiposity distribution (PC1) and socioeconomic status (SES) were assessed using principal component analysis. An analysis of covariance and Tukey’s post hoc test for unequal samples were performed to assess the effect of exposure to a natural disaster on the PC1, controlling for age, sex, Z-BMI, and SES. Prenatally and postnatally Aila-exposed children revealed a significantly more central-oriented pattern of relative subcutaneous fat distribution compared to the controls (*p* < 0.05). Early-life exposure to a natural disaster was related to an adverse pattern of relative adipose tissue distribution in preadolescent children.

## 1. Introduction

Environmental factors, especially the adverse conditions affecting an organism during its early, critical stages of development, may result in permanent alterations of particular anatomical structures or their functioning [1]. According to the “fetal origin of disease” hypothesis, fetal adaptation to a poor environment could program metabolic functions and lead to adverse health outcomes later in life, such as obesity, cardiovascular disease, and metabolic syndrome [2]. Animal models demonstrated that adverse conditions experienced by the pregnant female (e.g., nutrient restriction, stress) alter fetal programming, most probably via the impact of maternal glucocorticoids that pass through the placenta and alter the developing hypothalamic–pituitary–adrenal (HPA) axis [3], which has been linked to the regulation of energy balance and body composition [4].

In humans, the possibility of conducting such studies is often provided by spontaneous stressful situations, such as divorce, loss of job, or natural disasters (NDs) [5]. While the first ones are not “independent stressors”, as they are often under the personal influence [6], an ND is a spontaneous and sudden environmental phenomenon, randomly affecting a large number of individuals [7]. Therefore, the use of an ND in studies on early human development enables the elimination of the limitations related to the individual’s genetic or medical risk factors [6].

Events such as an ND may also be significantly harmful during infancy, when rapid growth potentially programs future disease risk [8], indirectly via obesity or adverse body composition [9]. Infant growth is an important predictor of subsequent body composition and truncal fat distribution in children [10,11], while excess truncal subcutaneous fat correlates with, e.g., insulin resistance [12]. What is important is that fat tissue distribution seems to be more strongly related to cardiometabolic risk factors than the body mass index (BMI) [11,13,14], and central adiposity is a significant predictor of future adverse health outcomes when the BMI is also normal [14].

Previous research revealed that in high-income (developed) countries, prenatal exposure to an ND resulted in an increased body mass in later life [7,15], whereas in a low-income (developing) country (India), the effect was opposite [16]. Regarding body fat distribution, based on high-income countries, results are not conclusive. For instance, elevated prenatal maternal stress (PNMS) related to an ND in Canada resulted in higher childhood central adiposity [6,17], while in the USA, it showed no effect [7]. In low-income countries, this relationship has not been studied so far. This is particularly interesting in the context of the opposite effect of an ND on the children’s weight status in developing vs. developed countries [16]. In developing countries, intrauterine growth retardation and poor linear growth during childhood are common due to insufficient diets and high rates of infection [18]. In an energy-restricted environment in remote rural areas in countries such as India, individuals may have different capabilities to cope with disaster-related hardships compared to the inhabitants of Western countries. It is also not known how the exposure to an ND during infancy could affect body fat distribution later in life. Therefore, the aim of this study was to assess the long-term effect of the exposure to an ND during prenatal or postnatal (infant) growth on the relative distribution of the subcutaneous adipose tissue in preadolescent Indian children compared to the control group.

## 2. Materials and Methods

### 2.1. Cyclone Aila and the Study Area

The study was conducted in the eastern part of India (West Bengal). Tropical cyclone Aila took place in West Bengal between 23 May and 26 May 2009. It was classified as a severe cyclonic storm, which had brought profound environmental destruction (for more details, see [19]). As a result of the cyclone, 138 people died, and a vast number of individuals lost all their properties (for more information, see [20]). The studied population lived in the Sundarban Delta, a region most affected by Aila (for detailed information on the study areas and the cyclone, see [21]). Inhabitants settled in these areas were rural people living on rain-fed, mostly mono-crop agriculture, forest products, and small-scale fishing.

The two most Aila-affected islands in the Sundarban Delta were selected: Satjelia and Kumirmari, under the community development block (CDB) Gosaba (district: South 24 Parganas). The destruction caused by the cyclone was, in general, evenly distributed across the villages. Most of the houses were destroyed, and all the agricultural lands and almost all domestic animals were lost. The control group was obtained from the neighboring rural areas of the eastern part of the broadly adjacent district, North 24 Parganas, under the CDB Bongaon. This region was not directly affected by Aila, except for a mild storm common for the season.

### 2.2. The Participants and Sampling Procedure

Each school from the Aila-affected areas was requested to participate in this study. Data on children exposed to Aila were collected from 22 schools (out of total 30) in Satjelia and all the 13 schools in Kumirmari. All children who attended the primary schools on these islands and met the recruitment criteria (as described below) were included in the study. A control group was recruited from 21 primary schools (out of total 34) in the adjacent district in a simple random procedure of sampling. Children having any kind of diseases or physical deformity which could affect measurements were excluded. The study sample did not include twins or multiple births. Informed consent from the mother/legal guardian of each child was obtained before the examination.

Recruitment criteria were as follows: prenatally Aila-exposed group—children exposed to cyclone Aila in utero (born between June 2009 and February 2010); postnatally Aila-exposed group—children exposed to cyclone Aila during infancy (born during the period less than 2 years preceding Aila); control group, non-exposed to Aila—children being in utero during cyclone Aila, living in the neighboring area not affected by Aila (born between June 2009 and February 2010.)

Out of the 987 children initially identified as a target for this study, 931 were present in the school during the study visits (56 were absent due to unknown reasons). However, 4 of them were sick and thus not measured, important information to be obtained from parents was not available for 37 children, and dates of birth of 22 children were later found incorrect, thus these children were excluded from the study. Consequently, data on 868 children constituted the final database.

Based on the variables required, the present study included 832 children (422 boys and 410 girls) aged 7.5–10.5 years (mean age = 8.5, SD = 0.55). Participants were divided into three groups: prenatally Aila-exposed (N = 336: 175 boys, 161 girls), postnatally Aila-exposed (N = 212: 105 boys, 107 girls), and control group (N = 284: 142 boys, 142 girls).

### 2.3. The Measurements and Socioeconomic Data

All measurements were obtained by trained field investigators following the standard measuring protocol. One of the simplest methods to evaluate body fat is the measurement of skinfold thickness [22]. Subscapular and suprailiac skinfolds are considered good indicators for central (truncal) adiposity and are commonly used for its assessment [12,22,23,24]. Central skinfolds are highly correlated with visceral fat or intra-abdominal adipose tissue and subcutaneous abdominal adipose tissue [24,25]. To assess the proportion of truncal adiposity relative to peripheral adiposity, 4 skinfolds were measured: subscapular (SSF) and suprailiac (SISF) skinfolds for truncal adiposity, and triceps (TSF) and biceps (BSF) skinfolds for peripheral adiposity. Skinfold thicknesses were measured to the nearest 1 mm using a skinfold caliper on the non-dominant arm. TSF and BSF were taken at the same level over the triceps and biceps muscles, respectively, in a halfway position between the acromion and the olecranon processes, with the arm hanging relaxed; SSF was measured below the inferior angle of the right scapula, and SISF was measured 1 cm over the iliac crest at the midaxillary line. Body height (in cm) and weight (in kg) were measured by anthropometer and weight scale, respectively. Body mass index BMI (kg/m^2^) was calculated and standardized for age according to LMS parameters (L for the skewness, M for median value, and S for the generalized coefficient of variation), separately for both sexes (based on CDC-2000 growth reference) [25].

Information on socioeconomic characteristics of families was collected from the mothers/legal guardians using questionnaire. All subjects inhabited rural settlements. Socioeconomic information included years of mother’s and father’s education, the total number of family members in a given household, and an average family income per month. Children’s birth weight was reported by the mothers.

### 2.4. Statistical Analyses

Estimation of relative body fat distribution was based on the ratios and principal components analysis (PCA) of the skinfolds, based on several previous studies (e.g., [26,27,28]). Four ratios (one for each skinfold) were calculated using following equation: log(one skinfold/sum of skinfolds) [27]. The ratios adjust for variation in overall subcutaneous fatness. PCA of these 4 ratios was performed to derive components that express the maximum contrast among them and reflect the trunk vs. peripheral contrast, if any. While the first component (PC1) presented a clear contrast between extremity and trunk, the second component (PC2) did not reveal any clear pattern. Moreover, analysis of covariance revealed no significant effect of age (F = 0.67, *p* = 0.42), Z-BMI (F = 0.55, *p* = 0.46), SES (F = 3.50, *p* = 0.06), sex (F = 0.03, *p* = 0.86), and groups (F = 2.38, *p* = 0.09) on PC2. Therefore, only the scores of the first component (PC1) were used in the further analysis as a proxy for relative body fat distribution; the lower the PC1 score values, the more central-oriented the pattern of the relative subcutaneous fat distribution.

PCA was also used to calculate the general socioeconomic status (SES) of participants. Loadings of the socioeconomic indices were as follow: father’s education, 0.47; mother’s education, 0.49; family size, −0.16; monthly family income per capita, 0.33. Scores of the first component, which explained 42% of the variance with eigenvalue 1.7, were used as a proxy for SES. The higher the score value of the first component, the higher the socioeconomic status.

The three study groups differed in terms of age (H(2, N = 832) = 552.79, *p* < 0.05), SES (H(2, N = 724) = 33.30, *p* < 0.05), and Z-BMI (H (2, N = 829) = 161.42, *p* < 0.05; see also [16]: prenatally and postnatally Aila-exposed children had significantly lower Z-BMI compared to the controls). Therefore, these variables were included in further analyses. The study groups did not differ in birth weight (H(2, N = 642) = 2.91, *p* > 0.05); thus, this variable was excluded from further analyses. General linear model (GLM) method using analysis of covariance (ANCOVA) was run with PC1 as dependent variable, sex and group as qualitative (categorical) independent variables, and age, Z-BMI, and SES as quantitative (continuous) independent variables (standardized residuals had a normal distribution; Levene’s test revealed homogeneity of variance: *p* > 0.05 for all variables). Statistical significance of all analyses was assumed at the level of *p* < 0.05. The effect size was assessed by the values of partial eta square (partial η^2^). All calculations were performed in Statistica 13.1.

## 3. Results

The descriptive statistics of the anthropometric indices are presented in Table 1.

The results of the PCA of the four skinfolds are summarized in Table 2. The positive loading of the PC1 for the triceps and biceps skinfolds and the negative loading for the subscapular and suprailiac skinfolds suggest a trunk–upper extremity contrast in the relative subcutaneous fat distribution. The lower the PC1 scores values, the more central-oriented the pattern of the relative subcutaneous fat distribution.

**Figure 1:**
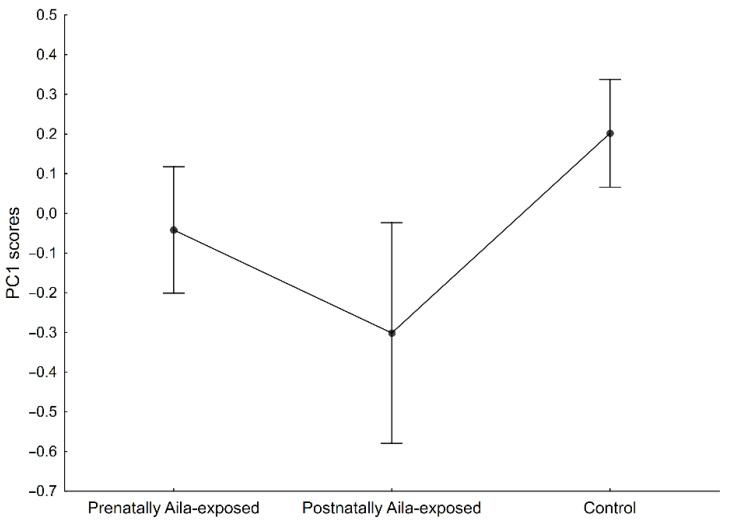
Differences in mean PC1 scores (a proxy for relative body fat distribution) between three studied groups. The lower the PC1 scores values, the more central-oriented the pattern of the relative subcutaneous fat distribution. Significant differences: prenatally Aila-exposed and controls (*p* < 0.05), and postnatally Aila-exposed and controls (*p* < 0.05).

The results of the ANCOVA (F = 2.41, *p* < 0.05) are presented in Table 3. No significant effect of sex, age, Z-BMI, or SES on the PC1 were found (*p* > 0.05). Moreover, the interaction between the group factor and sex was insignificant (*p* > 0.05). Only the group factor significantly differentiated the PC1 (*p* < 0.001). The effect size was significantly the highest for the group factor (partial η^2^ = 0.019). Tukey’s post hoc test for unequal samples revealed significant differences between the prenatally Aila-exposed children and the controls (*p* < 0.05), as well as between the postnatally Aila-exposed children and the controls (*p* < 0.05). No significant differences between the prenatally and postnatally Aila-exposed children were found (*p* > 0.05) (Figure 1).

## 4. Discussion

The present study provided evidence for a long-term effect of the early-life exposure to an ND on the relative subcutaneous adipose tissue distribution in preadolescent Indian children. The results indicated that children exposed to an ND in utero or during infancy showed a significantly more central-oriented pattern of subcutaneous adipose tissue distribution compared to their non-exposed peers. Moreover, this study contributes to the previous research by revealing that even though preadolescent Indian children exposed to an ND in their early life had lower values of nutritional and weight status indicators, compared to the controls [16], they exhibited an adverse relative body fat distribution pattern.

Our results are in line with some of the previous research on the long-term effect of prenatal stress due to an ND on central adiposity in children [6]. However, most of the previous studies on this issue revealed a higher relative weight afterward and a greater risk of obesity in subsequent years in children prenatally exposed to an ND [6,7,17,29], except for the Aila study [16]. Note, however, that those studies (except the Aila study) were conducted in high-income countries, where conditions for the recovery of a decreased weight status were favorable, which, in combination with the changes of the metabolic regulations during fetal programming, could lead to excessive fatness (e.g., [17]). In contrast, the Aila study was conducted on a population from a low-income country, living in energy-restricted, poor rural areas, severely affected by the ND, where resources were probably not sufficient for nutritional recovery and, consequently, early-life programming (in terms of body mass), at least to some extent, had lost its significance [16]. Our current study shows that despite the lower values of weight and the nutritional status indicators of the Aila-exposed children, body fat distribution tends toward an unfavorable, unhealthy pattern. This is of particular concern, as central fat distribution is an important indicator of future adverse health outcomes also when the BMI is normal [14].

The possible mechanism underlying a different pattern of the relative subcutaneous adipose tissue distribution in children prenatally exposed to natural disaster-related stress can plausibly be attributed to fetal programming of the hypothalamic–pituitary–adrenal (HPA) axis. High levels of maternal stress hormones cross the placenta and alter the normal functioning of the fetal HPA axis, resulting in a tendency to central adiposity (e.g., [6,30,31]). Previous research indicated that increased fetal exposure to glucocorticoids resulted in an increase in the central distribution of fat, despite a reduction in body size and overall adiposity [30]. This finding corresponds with our results on higher relative central adiposity accompanied by lower weight and nutritional status indicators in children exposed to an ND. Another possible mechanism is related to epigenetics. Research revealed that methylation in the genes involved in Type I and II diabetes mediated the effects of PNMS due to an ND on children’s central adiposity and BMI [17]. The long-term effects of the early-life exposure to an ND could also be related to the resulting environmental damage, followed by infants’ undernutrition, which in turn may lead to a dysregulated metabolism and impaired insulin signaling, affecting the unfavorable distribution of adipose tissue [32]. Early stunting might predispose an individual to a more central distribution of adiposity at later ages [32]. However, this does not seem to apply to the prenatal period in the Aila study, as the studied groups did not differ in terms of birth weight. Because we do not have information on the subjects’ body size during infancy, we cannot link our postnatal results to undernutrition with certainty. Nevertheless, the linkage between neonatal and infant undernutrition and later metabolic dysregulation leading to adverse fat patterning in childhood seems plausible, as the study population was made up of rural disadvantaged people who suffered nutritional deprivation due to the aftermath of an ND as the agricultural lands and fisheries became completely sterile. 

To our knowledge, this is the first study on the long-term effects of early-life exposure to an ND on relative fat distribution conducted in a developing country, where significant geographic barriers, poor infrastructure, harsh living conditions, and insufficient health services make adverse events more difficult to cope with [33]. As mentioned above, previous studies on the prenatal exposure to an ND were carried out in countries with efficient emergency services and no deaths caused directly by the disasters. The damage caused by Aila was much more severe than those of the previous projects (Quebec Ice Storm, Queensland flood, Iowa floods), with more than a hundred human deaths, a massive loss of livestock, and the destruction of farmlands and households. An important advantage of this study is also a reasonably large sample size, delivered from rural communities, homogeneous in terms of socioeconomic living conditions, dependent on the natural environment, and having a similar lifestyle. This was an important precondition of sampling, as the differences in socioeconomic status could, to some extent, modulate the coping mechanisms against the disaster.

However, there are also some potential limitations to report. In this study, we did not take duplicate measures of skinfolds. Moreover, we did not measure waist circumference, which is considered a reliable surrogate for the amount of abdominal fat and visceral adiposity in adult populations [22,34,35,36,37,38]. Note, however, that in children, the correlations of intra-abdominal and subcutaneous adipose tissue with the SISF, SSF, and waist circumference are strong and closely similar [39]. Moreover, some research indicates that the skinfolds measurements are the best non-invasive technique in predicting subcutaneous as well as intra-abdominal fat before puberty, more pronounced than circumferences [40]. On the other hand, according to Goran and Malina [41], the ratio of the trunk to extremity skinfolds explained only 62% of the variation in the abdominal visceral fat in children. Yet, although skinfold thickness does not measure fat in accurate quantitative terms, it still provides a reliable index of the relative fat distribution [32], which was the crux of this study. Moreover, among specialized techniques for the assessment of subcutaneous body fat, skinfolds measurement showed better agreement with the results from computed tomography than an ultrasound [42]. Computed tomography is the most accurate and reproducible technique for body fat measurement, especially in terms of abdominal adipose tissue; however, this is also expensive, time-consuming, and involves exposure to ionizing radiation [43], while anthropometric measurements are relatively easy to perform and cheap [44]. They are valuable for classifying individuals according to different types of fat distribution and for general use in epidemiological studies [44]. Moreover, the principal components protocol reduces the influence of overall or absolute fatness on relative subcutaneous fat distribution [27]. Moreover, as calculating the principal components from raw skinfold data may bias the results toward the measurement with the greatest variance [27,45], the logarithms of ratios were used in our study. Thus, the problem of differences in variance between the measurements was avoided, and a measure of subcutaneous adipose tissue distribution was obtained independent of overall fatness [27]. Based on the above, the use of this anthropometric body fat measure as well as the method for assessing the relative subcutaneous fat distribution seems justified and appropriate.

## 5. Conclusions

Our study revealed that early-life exposure to an ND was related to an adverse pattern of relative subcutaneous adipose tissue distribution in preadolescent Indian children, despite their low BMI values. It seems that exposure to such unfavorable early-life conditions, followed by a subsequent central-oriented distribution of adipose tissue, may increase the risk of non-communicable diseases later in life. The adult pattern of relative fat distribution emerges during adolescence (e.g., [46]), and previous research has shown that the effects of natural disaster-related PNMS on central adiposity tend to increase as the children grow older [6]. Therefore, given the likely life-long effects of early-life exposure to an ND, particularly pregnant women and infants should be targeted for early disaster-related interventions, and the health monitoring of exposed children should be an important public concern.

## Figures and Tables

**Table 1:** Descriptive statistics of the anthropometric indices for the three study groups.

	Prenatally Aila-Exposed N = 336	Postnatally Aila-Exposed N = 212	Control N = 284
	Mean (SD)	Mean (SD)	Mean (SD)
Age (years)	8.1 (0.23)	9.3 (0.34)	8.3 (0.23)
Height (cm)	120.7 (5.06)	127.19 (5.98)	122.8 (5.45)
Weight (kg)	21.0 (3.07)	23.43 (3.72)	24.73 (4.88)
SSF (mm)	5.99 (2.23)	7.74 (3.32)	7.13 (3.89)
SISF (mm)	4.80 (2.29)	6.40 (3.48)	6.22 (4.30)
TSF (mm)	7.67 (2.76)	9.73 (3.75)	10.55 (4.29)
BSF (mm)	4.17 (1.69)	6.03 (2.95)	5.32 (2.90)

SSF—subscapular skinfold; SISF—suprailiac skinfold; TSF—triceps skinfold; BSF—biceps skinfold.

**Table 2:** Results of principal components analysis of the four skinfold thicknesses.

Quotient of Skinfolds	PC1	PC2
biceps	0.419	−0.532
triceps	0.830	0.242
subscapular	−0.474	0.770
suprailiac	−0.761	−0.509
Eigenvalue	1.668	1.194
% variance	41.7	29.8

**Table 3:** Results for ANCOVA, with PC1 (a proxy for relative body fat distribution) as dependent variable, and group, sex, age, Z-BMI, and SES as independent variables, as well as interaction between group factor and sex.

Effect	F	*p*	Partial η^2^
group	7.07	<0.001	0.019
sex	0.01	0.94	0.000
age	1.52	0.22	0.002
Z-BMI	1.64	0.20	0.002
SES	0.001	0.97	0.000
group × sex	0.35	0.71	0.001

## Data Availability

The data presented in this study are available on request from the corresponding author.
